# Comparison of anatomical visual features of the eyeball, lens, and retina the diurnal common kestrel (*Falco tinnunculus rupicilaeformis*) and the nocturnal little owl (*Athene noctua glaux*)

**DOI:** 10.1186/s12917-024-04371-7

**Published:** 2024-11-29

**Authors:** Walaa Shalaby, Ramadan Kandyel, Mohamed Abumandour, Fawzyah A. Al-Ghamdi, Doaa Gewily

**Affiliations:** 1 Zoology Department, Faculty of Science (girls), Al-Azhar University, Cairo, Egypt; 2 Department of Zoology, Faculty of Science, Tanta University, Tanta, Egypt; 3 Department of Biology, Faculty of Arts and Sciences, Najran University, Najran, Saudi Arabia; 4 Department of Anatomy and Embryology, Faculty of Veterinary Medicine, Alexandria University, Abees 10th, Alexandria, Egypt; 5 Department of Biological Science, College of Science, University of Jeddah, P.O. Box 80327, Jeddah, 21589 Saudi Arabia

**Keywords:** Nocturnal little owl, Diurnal kestrel, Visual adaptations, Retina, Histology, Transmission electron microscopy, Scanning electron microscopy

## Abstract

Our study aimed to compare the anatomical features of the eyeball, lens, and retina between the two raptor birds of different visual active clock hours: the diurnal common kestrel (*Falco tinnunculus rupicilaeformis*) and the nocturnal little owl (*Athene noctua glaux*) using gross, morphometric analysis, histological, and scanning and transmission electron microscopy techniques. The semi-spherical eyeball of the kestrel had less convexity on the anterior surface than on the posterior surface; meanwhile, it was relatively larger in the owl. There is a relationship between the corneal diameter (CD) and the eye axial length (AL). There were significant differences in the retinal layer thickness between the two raptors, in which the diurnal kestrel had a thinner pigmented epithelium and photoreceptor layers compared to the nocturnal owl. Moreover, the inner nuclear and outer nuclear, inner plexiform, and outer plexiform layers in the diurnal kestrel were larger than those in the nocturnal owl. The differences in the pigmented epithelium layer lead to the higher visual acuity and better color vision of the diurnal kestrel compared to the nocturnal owl. The photoreceptor layer in diurnal kestrel was composed of single large and double cones, which are composed of chief cones and accessory cones; meanwhile, the photoreceptor layer in nocturnal owl had only single elongated rods. We concluded that the lens and retina of the two raptors revealed great variations in reflecting the adaptation of each bird to different modes of life. The statistical analysis found a strong positive correlation between the axial length of the eye and the corneal diameter in both birds, as indicated by the Pearson correlation coefficient.

## Highlights

The semi-spherical eyeball of the kestrel had less convexity on the anterior surface than on the posterior surface; meanwhile, it was relatively larger in the owl. The kestrel had the lowest CD: AL ratios, while the owl had the largest CD: AL ratios.

The retinal thickness in the diurnal kestrel had a thinner pigmented epithelium and photoreceptor layers compared to the nocturnal owl. The inner nuclear and outer nuclear, inner plexiform, and outer plexiform layers in the diurnal kestrel were larger than those in the nocturnal owl.

The photoreceptor layer in diurnal kestrel is composed of single, large, and double cones; additionally, they are composed of chief cones and accessory cones; meanwhile, the nocturnal owl has only single rods.

The statistical analysis found a strong positive correlation between the axial length of the eye and the corneal diameter in both birds, as indicated by the Pearson correlation coefficient.

## Introduction

The carnivorous diurnal common kestrel (*Falco tinnunculus rupicolaeformes*) is a small predatory bird that naturally preys on numerous agricultural pests. Additionally, it belongs to the *kestrel* subfamily, the *Falconidae* family, and Genus *Falco*. Old World Kestrel is also named Eurasian Kestrel and European Kestrel [1]. Geographically, this species is distributed throughout North Africa, with an emphasis on Egyptian wildlife. Common kestrels eat large insects, especially beetles and grasshoppers, as well as rodents such as voles and mice [2]; additionally, they sometimes consume earthworms, little birds, reptiles, lizards, and snakes [3]. The nocturnal little owl (*Athene noctua glaux*) lives mostly in temperate and tropical regions of Europe and North Africa [1]. They belong to the genus *Athene* and the family *Strigidae*, which includes true or common owls. They consumed insects, earthworms, other invertebrates, tiny vertebrates, and other food items. The *Athene noctua glaux* (*Savigny*,* 1809*) primarily lives along the coasts of North Africa and southwest Israel [4].

The eye is one of the body’s major sensory organs and was crucial for communication between living things and their environment [5–7]. Many characteristics of vertebrates’ eyes were adaptations to the visual environments in which they have evolved [8–10]. Eye size differed widely among vertebrates and was closely related to visual ability [11]. Greater visual acuity, increased light sensitivity, or combinations of both are all benefits of having large eyes. In order to provide a usable code for additional visual information processing, eyes replicate images on the retina [11].

The size of an animal’s eye had a significant impact on its visual ability [11]. Larger eyes provided longer focal lengths, which affect the size of the retinal area where the image of an object extends [11]. Because of this, the acuity of the eye was directly correlated with the size of the eye and the diameter of the lens. The diurnal kestrels and nocturnal owls had relatively larger eyes than other flying birds, and predatory raptors had larger eyes than species that consumed carrion [12, 13]. Philippine eagles, Golden eagles, and Secretary birds have large raptor eyes over 30 mm in axial length, while *Microhierax fringillarius* and *Microhierax caerulescens* have smaller eyes less than 10 mm [14]. However, other parameters like the angular spacing of the receptors, the caliber of the optic components, and the spacing between ganglion cells also had an impact [8, 15].

All vertebrates, including birds, had eyes similar to cameras, with the cornea, aqueous humor, lens, and vitreous humor absorbing light before reaching the retina [8]. The lens was crucial for mammalian adaptation, but evaluating its optical characteristics during accommodation was challenging due to its internal location [16]. The retina, a fundamental structure in all vertebrates, varies significantly in visual requirements among species [17, 18]. Birds, particularly birds of prey, have distinctive retinal architecture, with differences in shape, areas of greatest visual acuity, and vascularization, despite having the same arrangement of retinal layers [19]. Vertebrates, including raptors, had an inverted retina, consisting of two layers: a pigmented layer with a single row of cuboidal epithelium and nine neural layers with photoreceptors and different neurons [8, 20]. Light travels through these layers before reaching the outermost layer, the photoreceptors. Bird lenses have two compartments: the main lens body and an annular pad separated by a *cavum lenticuli* [21]. Avian lenses have an annular pad surrounding their central core, distinguishing them from mammals [22]. The annular pad is separated by a fluid chamber called the *vesicula lentis* [21].

The anatomical arrangement of the visual photoreceptor layer may be a hydrostatic mechanism that transfers pressure from the ciliary muscle to the central core [18, 22–24]. Other cells, including bipolar, horizontal, glial, and ganglion cells, also function as photoreceptors with non-visual functions [25]. These photoreceptors consist of an inner synthetic area connected to an outside light-sensitive area via a nuclear region, synaptic termination, and non-motile joined cilium [26]. The two types of retinal photoreceptors, rods and cones, were identified based on their microscopic appearance [18]. Vertebrates’ optical cells, either rods or cones, are linked to scotopic or photopic vision, with the discrepancy attributed to morphological differences in photoreceptor structures [9, 18, 23, 24, 27, 28]. Most vertebrates have duplex retinae, with rods and cones found together. The ratio of rods to cones varies based on habits and surroundings [29, 30]. Most rods are found in nocturnal species’ retinae, while most cones are found in diurnal species’ retinae [27, 31]. Diurnal animals have a larger cone-to-rod ratio, resulting in cone-rich retinas and better visual acuity in birds. However, nocturnal birds have a higher density of rod photoreceptors, indicating sensitive vision [18, 32].

The study aimed to compare the anatomical features of the eyeball, lens, and retina between the two raptor birds of different visual active clock hours: the diurnal common kestrel (*F. tinnunculus rupicilaeformis*) and the nocturnal little owl (*A. noctua glaux*) using gross, morphometric analysis, histological, and scanning and transmission electron microscopy techniques. This study was designed to understand the visual adaptations to these different lifestyles, whereas the diurnal falcon had highly visual acuity during the daytime and the nocturnal owl had highly sensitive vision during the night. Finally, we compared our findings to information that had previously been published on the eyeball, lens, and retina of the different feeding lifestyles with different visual activities.

## Materials and methods

### Birds’ collection and preparation

Seven adult Egyptian endemic two carnivorous bird species with different visual active clock hours: the nocturnal little owl (*A. noctua glaux*) and the diurnal common kestrel (*F. tinnunculus rupicilaeformis*) of 1200 to 1400 g in weight were collected from the local pet shops in Alexandria city, Egypt. All birds were transported in travel pet cages within 2 h to the animal housing of the Department of Zoology, Faculty of Science (Girls), Al-Azhar University, Cairo, Egypt, to be examined by a veterinarian to ensure that they were healthy and free from any abnormalities or injuries, especially in their eyes. For further examination of the eyes, both carnivorous species, the common kestrel and the little owl, were first anesthetized with 2 mg/kg of xylazine intramuscularly [33] and then euthanized with a lethal dose of ketamine (100 mg/kg) intramuscularly, unconscious and painless. A ketamine injection resulted in brain death 90 s after injection, confirmed by the absence of reflexes, movement, heartbeat, and cardiacelectrical activity. The study found that despite rapid death, no histopathological changes were observed in the tissue specimens studied [34, 35], and all birds were decapitated after profound narcosis.

This study followed the guidelines established for the ‘Sampling protocol for the pilot collection of catch, effort, and biological data in Egypt’ [36]. The enucleated eyes were carefully extruded from the lens and retina and immersed in the fixed formalin solution at 10% for 6 h. Then, the extirpated lens and retina were transferred to the anatomical lab to prepare for the histological and electron microscope techniques after the gross morphological examinations occurred under the stereomicroscope. The anatomical terms were applied according to *Nomina Anatomica Avium* [37].

### Anatomical shape and gross measurements of the eye

Each eyeball was cleaned from all fascia and extra-ocular muscles before being inflated by injecting a small amount of formaldehyde 10% in 0.1 M PBS with a syringe and small-gauge needle [38]. The fixative was injected into the eyeball until it was fully inflated and would not accept any more liquid. All 20 eyes could be fully inflated, and so they were used for subsequent measurements. Maximum CD and maximum eye AL were measured to the nearest 0.01 mm using digital calipers. These values were used to calculate the eye shape, which was defined as the common logarithm (log 10) of the CD: AL ratio [38].

### For gross stereomicroscopic observations

Four lenses and retinas from both birds were examined grossly under the stereomicroscope (*Olympus VM VMF 2x*,* Eyepiece 10x Stereo Microscope*, *Japan*) to describe their anatomical features, location, and shape. Our gross anatomical features were photographed by the Olympus Plus camera (*Olympus*, *Tokyo*, *Japan)*.

### For morphometric measurements of retina

The thickness of the whole retina and its layers in the investigated raptors were measured by linear ocular micrometers. The ratio of the relationship between the outer and inner nuclear layers was investigated [39] to determine the diurnal and nocturnal patterns of the birds. The measurements were taken by image pro plus program.

### For histological examinations

Four lenses and retinas from both carnivorous birds were used to demonstrate their histological properties [18, 40]. Four section for each specimen and each section took approximately six measurements in different regions. The examined eyeballs’ specimens of both birds were fixed in Bouin’s solution for 4 h, cut in cross-sectional acute scalpel to 1/3 and 2/3, then let go in Bouin’s solution for 10 h at room temperature, followed by washing 24 h with 70% ethyl alcohol, dehydrated in ascending grades of ethyl alcohol, cleared in xylene, and embedded in molten paraffin wax at 58–62 °C. The collected specimens (0.5 cm^3^) were immersed in 10% neutral buffered formal saline and then they were transferred to 70% alcohol after 48 h. The tissue samples were dehydrated in an ascending grade of ethanol, cleared in xylene, impregnated, and embedded in paraffin wax. Sections of 5–6 μm serial paraffin sections were cut using a Leica rotatory microtome (*RM 20352035; Leica Microsystems*,* Wetzlar*,* Germany*) and mounted on glass slides. Paraffin sections were organized and stained with Hematoxylin and Eosin (H&E) stain for routine histological technique [41].

### *For* statistical comparison analysis between the retinal layer thickness between the diurnal common kestrel and the nocturnal little owl

The obtained SEM images were processed using the ImageJ 1.53 k application (National Institutes of Health, United States). In this analysis, we used Welch’s t-test to compare the mean of various parameters between the diurnal common kestrel and nocturnal little owl datasets. Welch’s t-test was chosen because it does not assume equal variances between the two groups, providing a more accurate assessment of potential differences. For each parameter, we calculated the t-statistic and p-value to determine if the differences were statistically significant. To account for multiple comparisons, we applied the Bonferroni correction, adjusting the significance threshold to control for Type I errors. Parameters with corrected p-values below 0.05 were considered significant.

### For scan electron microscopy investigations

Three lenses and retinas from both birds were examined under the SEM to demonstrate the ultrastructural nature of the lens and retinal surface [40]. The samples were fixed in 2% formaldehyde and 1.25% glutaraldehyde in 0.1 M sodium cacodylate buffer, pH 7.2, at 4 °C. Once fixed, the samples were washed in 0.1 M sodium cacodylate containing 5% sucrose, processed through tannic acid, and finally dehydrated in an increased concentration of ethanol (50, 70, 80, 90, 95, and 100%) for 15 min in each concentration. The samples were then critical point dried in carbon dioxide, attached to colloidal carbon stubs, and sputtered with gold and palladium. Specimens were examined and photographed with a JEOL-SEM operating at 15 kV at the Faculty of Science, Alexandria University, Egypt.

### Transmission electron microscopy investigations

Three lenses and retinas from both birds were examined under the TEM to demonstrate the cellular structure of the lens and retinal surface [9]. The retina was carefully removed from the eye cup and separated from the choroid and vitreous body using forceps. Each retina was cut into small pieces and fixed in 2.5% glutaraldehyde in 0.1 M cacodylate buffer (pH 7.4) for about five hours, followed by washing in phosphate buffer (pH 7.4) and post-fixation in buffered 1% osmium tetraoxide. The specimens were washed thoroughly in buffer, dehyderated in an ascending series of cold ethyl alcohols, cleared in propylene oxide, and mounted in epoxy resin. The retina was cut into 1 mm3 pieces. The samples were immediately fixed in the same fixative solution, pH 7.4, at 4 °C for 6 h [42]. Tissues were washed in cold (4 °C) 0.1 M phosphate buffer every 15 min for 2 h after initial fixation. Then, the samples were rapidly dehydrated through increasing concentrations of ethanol, transferred to propylene oxide, and placed overnight in a 1:1 mixture of propylene oxide and epoxy araldite [43]. Semi-thin Sect. (1 mm) were first cut, stained with toluidine blue, and viewed with light microscopy. Ultrathin Sects. (60–100 mm) were then cut with a glass knife with an L.K.B. microtome and stained with uranyl acetate, followed by lead citrate [43]. The ultrathin sections were examined with a Jeol transmission electron microscope operating at 100 Kv at the Faculty of Science, Alexandria University.

## Results

### Morphological anatomy and morphometric analysis

#### Gross morphometric analysis of Eyeball

**Fig. 1:**
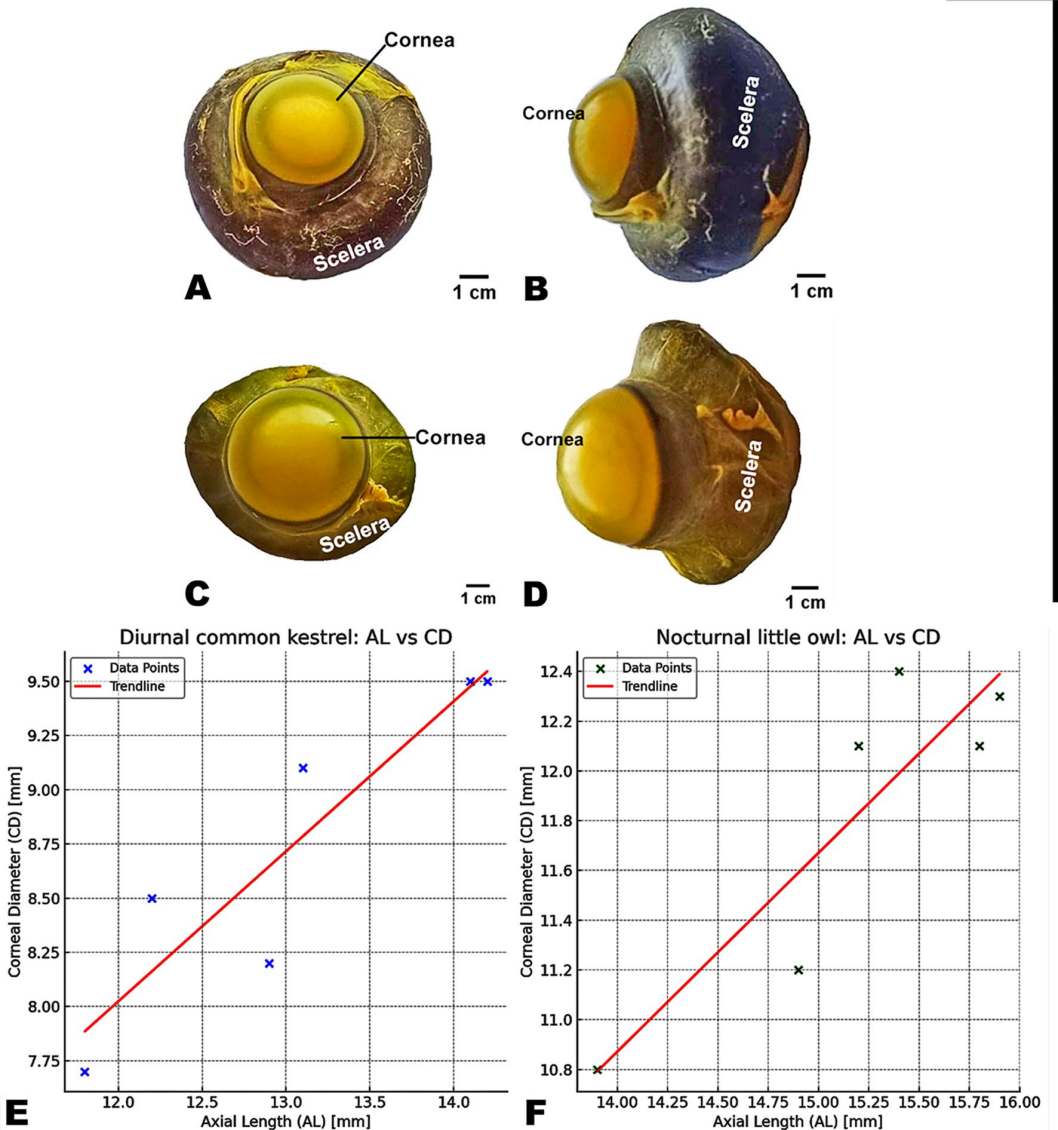
Anatomical Dorsal (Views **A** and **C**) and lateral (Views **B** and **D**) images of excised eyeballs, and Statistical Comparison between the axial length (AL) and corneal diameter (CD) (Views **E**-**F**) of the diurnal common kestrel (***F***. *tinnunculus rupicilaeformis*) and the nocturnal little owl (***A***. *noctua glaux*), respectively. Each eyeball has been fully inflated with fixative, as described in Materials and Methods, allowing eye AL and maximum CD to be measured. Scale bars represent 10 mm. In both species, the axial length of the eye and the corneal diameter are highly correlated

**Fig. 2:**
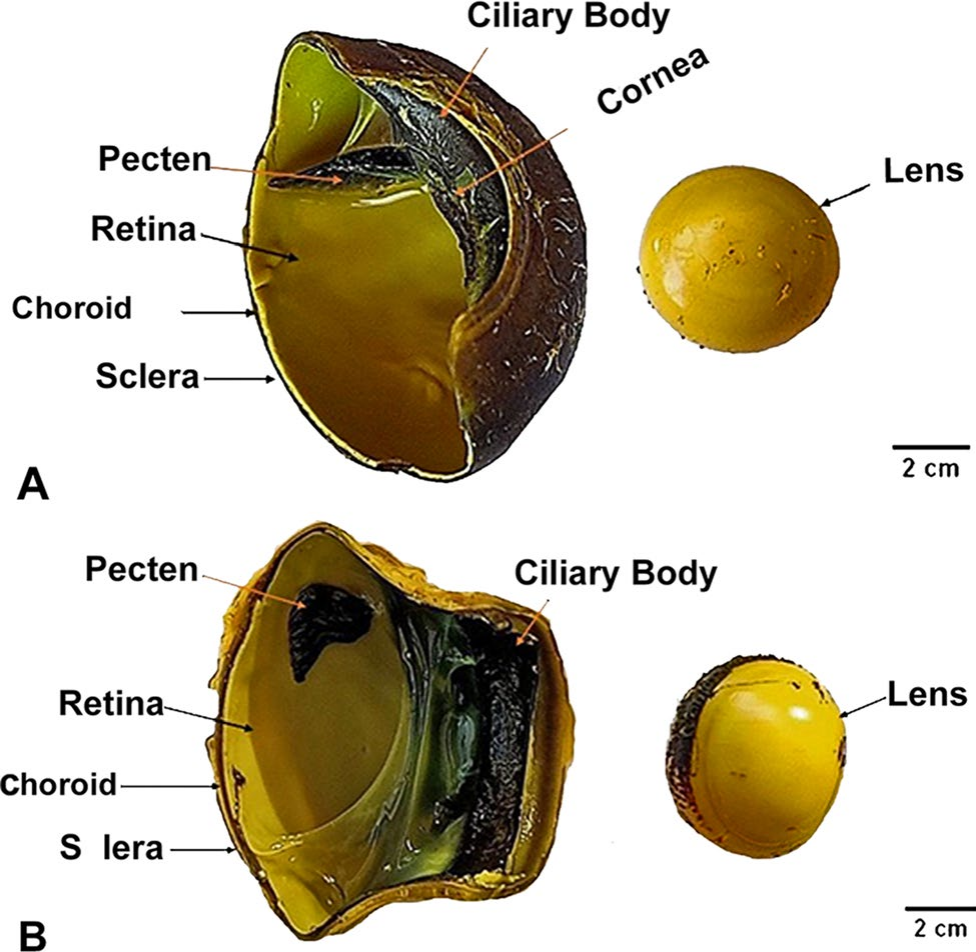
Anatomical image describing the internal structures of the eyeball of the diurnal common kestrel (View **A**) and the nocturnal little owl (View **B**)

The eyeball of the diurnal common kestrel (*F. tinnunculus rupicilaeformis*) was semi-spherical, with less convexity on the anterior surface than on the posterior surface **(**Figs. 1A and 2A**).** The eye lens was less convex on the anterior surface than the posterior one. The average diameter of the lens was 0.5 ± 0.14 cm, and its average thickness (anterior-posterior) was 0.42 ± 0.075 cm. Meanwhile, in the nocturnal little owl (*A. noctua glaux***)**, the eyeball was relatively larger than the eyeball of the diurnal common kestrel, with elongated eye tubes or cylinders in the shape and an end with a flattened disc **(**Figs. 1B and 2B**).** The eye lens was more convex on the anterior surface than the posterior one. The lens was more strongly curved on its corneal surface than on its vitreal surface. The average diameter of the lens was 1.1 ± 0.26 cm, and its average thickness (anteriorposterior) was 0.52 ± 0.12 cm, as described in **(**Table 1**)**.

**Table 1:** Average diameter of the lens (DL) and thickness (both measured in cm) for two species of raptors: the diurnal common kestrel (*F. Tinnunculus Rupicilaeformis*) and the nocturnal little owl (*A. noctua glaux*)

species	Average DL ± SD	average thickness ± SD
Diurnal common kestrel	0.5 ± 0.14	0.42 ± 0.075
Nocturnal little owl	1.1 ± 0.26	0.52 ± 0.12

#### Shape, measurements, and statistical comparison analysis between the eye layers between the diurnal common kestrel and the nocturnal little owl

This study examined the relationship between corneal diameter and axial length of the eye as an eye shape, defined here as the ratio of corneal diameter (CD) to eye axial length (AL). Eye shape varied considerably among the species examined, as described in **(**Table 2**and** Fig. 1**).** The diurnal common kestrel had a noticeably smaller cornea diameter (CD) relative to eye axial length (AL), which had the lowest CD: AL ratios of -0.175. In contrast, the nocturnal little owl occupies one end of the spectrum with the largest cornea diameter (CD) relative to eye axial length (AL), as described in (Table 2) and an average log 10 (CD: AL) ratio of -0.109.

In both species, the axial length of the eye and the corneal diameter are highly correlated. In the diurnal common kestrel, the Pearson correlation coefficient is **0.91**, which indicates a very strong positive correlation between AL and CD. This means that as the axial length of the eye increases, the corneal diameter tends to increase as well **(**Fig. 1E **and** Table 2**)**. The p-value of **0.011** suggests that this correlation is statistically significant, meaning there is less than a 1.1% probability that this relationship is due to random chance. In the nocturnal little owl, the Pearson correlation coefficient is **0.89**, also indicating a strong positive correlation between AL and CD **(**Fig. 1F **and** Table 2**)**. Like in the kestrel, a longer axial length corresponds to a larger corneal diameter **(**Fig. 1E-F **and** Table 2**)**. The p-value of **0.017** suggests this relationship is statistically significant, with less than a 1.7% probability of it being random.

**Table 2:** Eye AL, maximum CD (both measured in mm) and eye shape (shape) values for two species of avian raptors: the diurnal common kestrel (*F. Tinnunculus Rupicilaeformis*) and the nocturnal little owl (*A. noctua glaux*)

Species	Eye1	Eye 2	Eye 3	Eye 4	Eye 5	Eye 6	Average ± SD
Diurnal common kestrel							
AL	14.1	12.2	11.8	12.9	14.2	13.1	13.05 ± 0.97
CD	9.5	8.5	7.7	8.2	9.5	9.1	8.75 ± 0.74
Shape	-0.174	-0.161	-0.187	-0.194	-0.174	-0.161	-0.175 ± 0.013
Nocturnal little owl							
AL	15.9	14.9	15.2	15.4	13.9	15.8	15.2 ± 0.73
CD	12.3	11.2	12.1	12.4	10.8	12.1	11.8 ± 0.66
Shape	-0.114	-0.125	-0.102	-0.092	-0.107	-0.114	-0.109 ± 0.011

### Histological and scan Electron Microscope Investigation

#### Lens

**Fig. 3:**
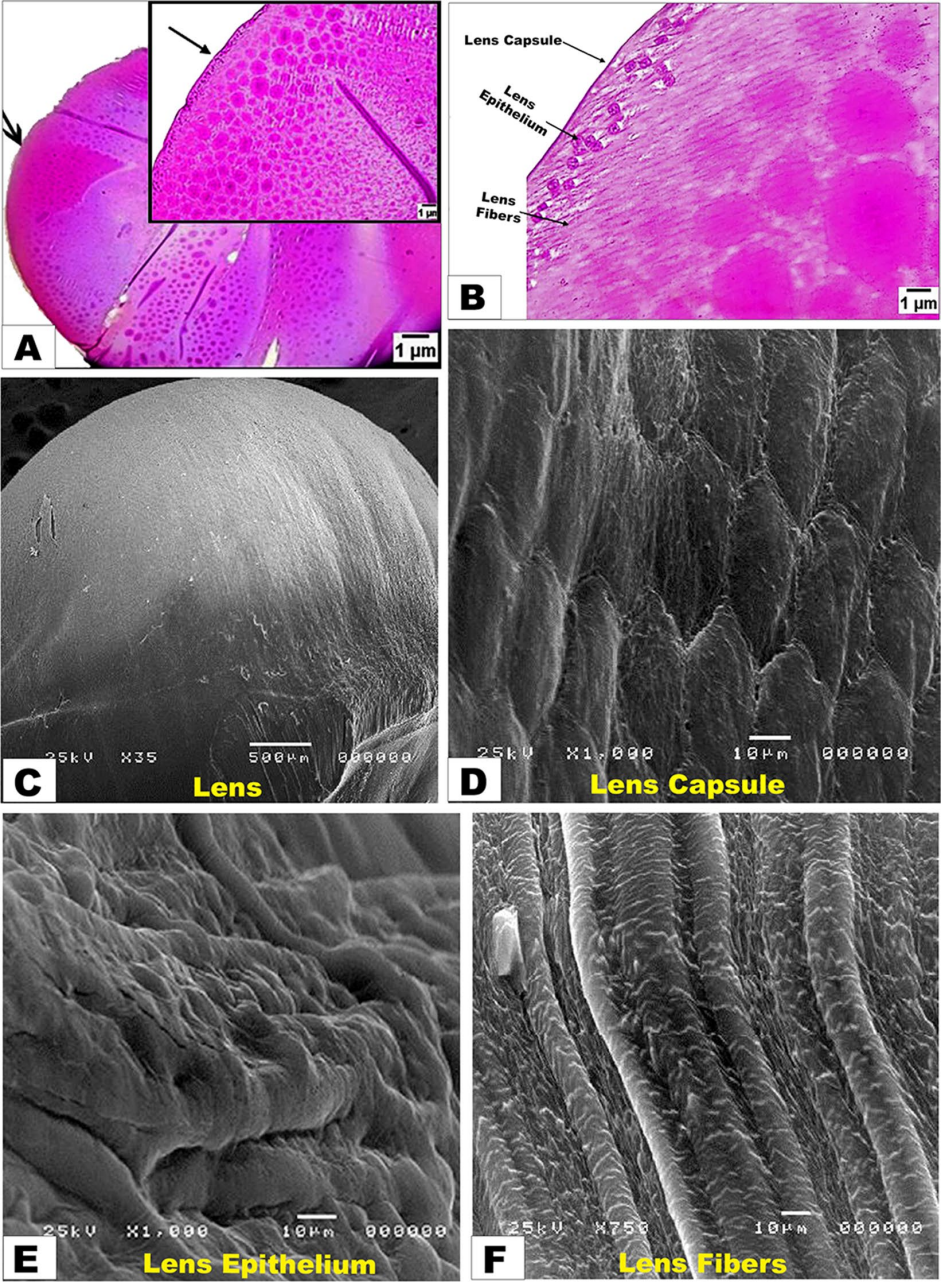
Photomicrograph images of the histological (Views **A** and **B**) and scanning electron microscopic (Views **C**, **D**, **E**, and **F**) features of the diurnal common kestrel lens

By LM examinations, the eye lens of diurnal common kestrel was covered by a thin capsule; additionally, at high magnification, the outer part of the lens capsule was less dense and consisted of fine fibers **(**Fig. 3A-B**).** The lens epithelium was situated in the anterior part of the lens between the capsule and the third part of the lens fibers, with squamous cells that had round nuclei surrounding the light cytoplasm, and then led to the third part of the lens fibers, which were observed parallel to each other **(**Fig. 3B**).** By the SEM investigation, the dorsal surface of the lens of the diurnal common kestrel exhibited the lens capsule (Cap) surrounding the lens, which seemed to have a hexagonal shape of fibers **(**Fig. 3C-D**).** The lens epithelium (Ep) was located under the lens capsule. Its surface is covered by a layer of epithelial cells; additionally, many folds appeared on the surface of the epithelium lens towards its surroundings **(**Fig. 3E**).** Then, the lenticular fibers appeared under the lens epithelium in a straight, folded arrangement **(**Fig. 3F**).**

**Fig. 4:**
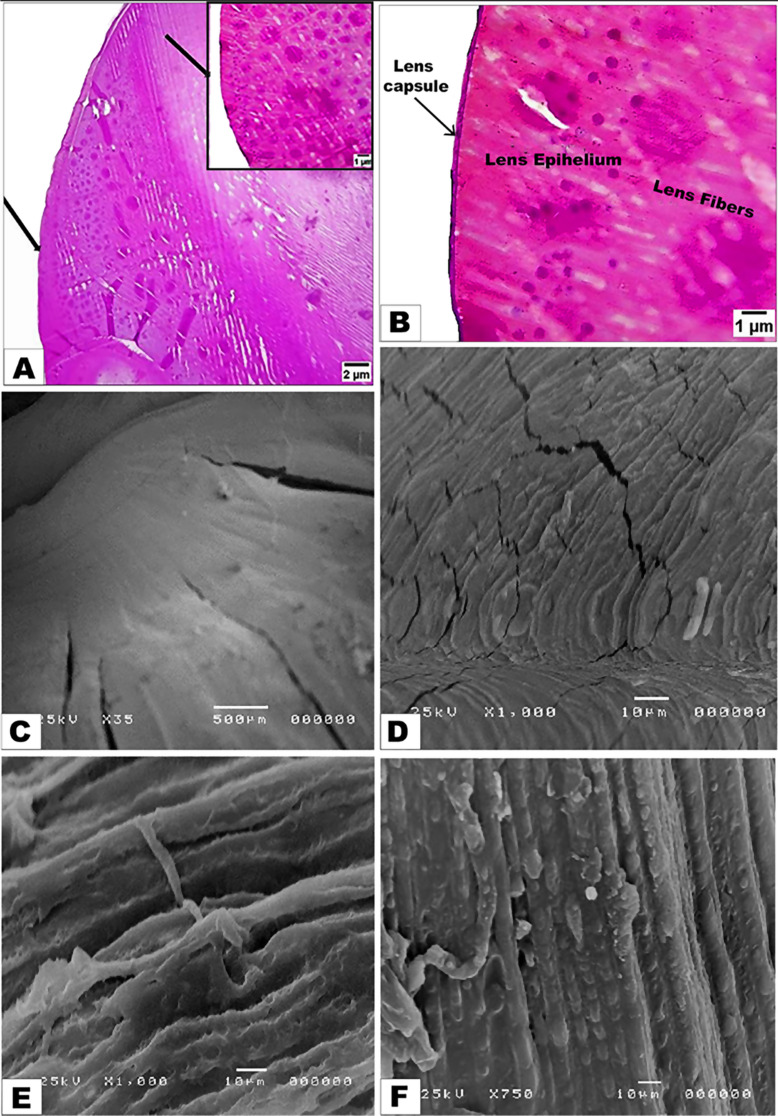
Photomicrograph images of the histological (Views **A** and **B**) and scanning electron microscopic (Views **C**, **D**, **E**, and **F**) features of the nocturnal little owl lens

By LM examinations, the eye lens of the nocturnal little owl was surrounded by a dense capsule (the outermost layer). With high magnification, the outer part of the lens capsule was denser with collagen fibers **(**Fig. 4A-B**).** The capsule was thinner at the posterior end of the lens. The lens epithelium was observed below the lens capsule, located between the lens capsule and the lens fibers, with layers of squamous cells. These cells had small, round nuclei **(**Fig. 4B**)** and the lens fibers were observed parallel to each other **(**Fig. 4B**).** Some of the fibers had a deep color, while others were light-colored. The lens fibers seemed to be thicker in the lateral areas than the middle ones. By the SEM investigation, the lens surrounded by a capsule (Cap) of fibers. Its anterior surface under the lens capsule is covered by a monolayer of epithelial cells (Ep) **(**Fig. 4D**).** Additionally, many folds appeared on the surface of the epithelium lens towards its surroundings **(**Fig. 4E**).** The majority of fiber cells were elongated, straight-folded, appeared in cross-sections, and were very regularly organized in closed sheets **(**Fig. 4F**).** The lens epithelium, thicker around the equator, forms the annular pad, a nucleated fiber feature found in both bird lenses, surrounded by a thick lens capsule.

#### Retina

**Fig. 5:**
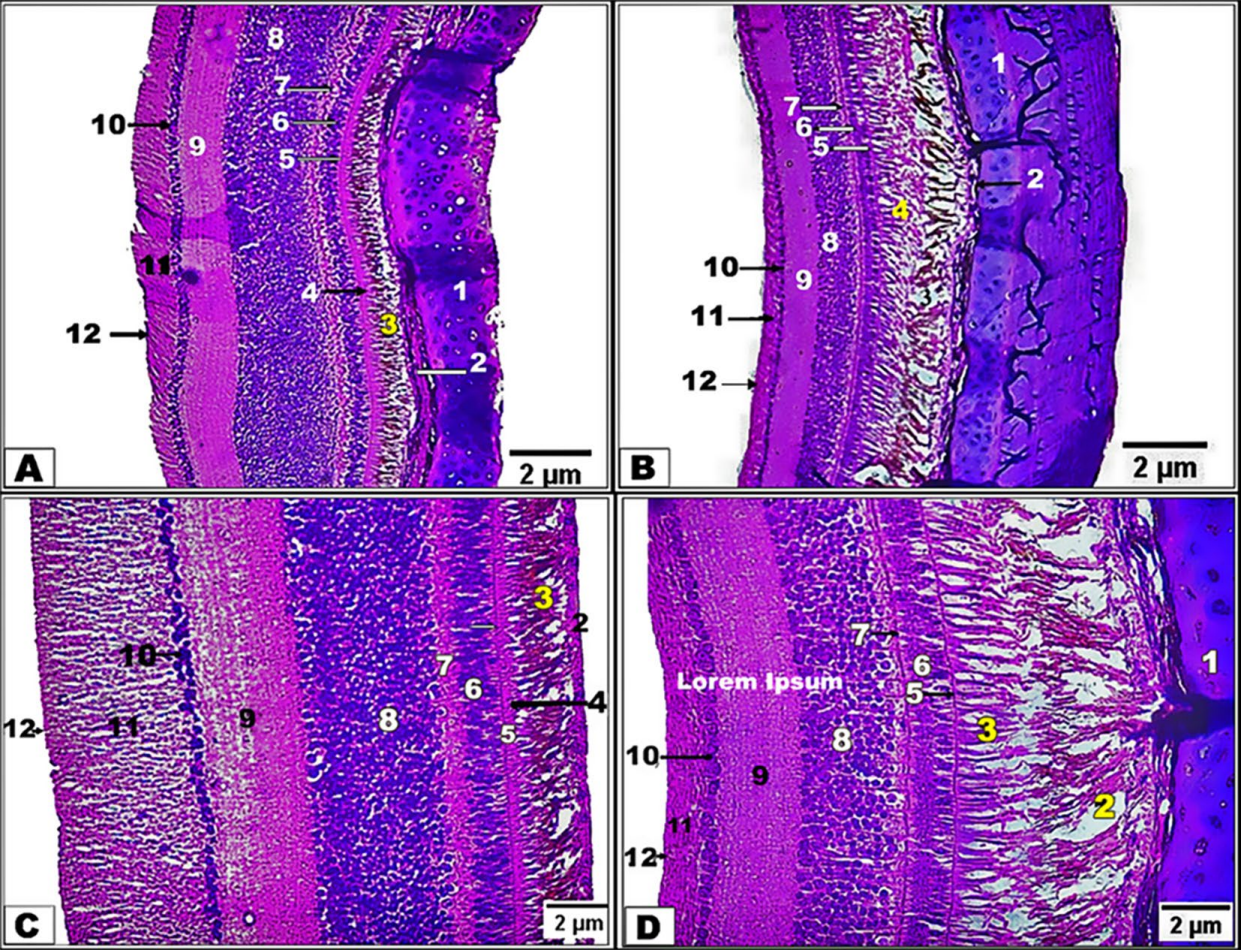
Photomicrograph of the retina in the diurnal common kestrel (Views **A** and **C**) and the nocturnal little owl (Views **B** and **D**) showing the sclera **(1)**, choroid **(2)**, pigmented epithelium **(3)**, photoreceptor layer **(4)**, outer limiting membrane **(5)**, outer nuclear layer **(6)**, outer plexiform layer **(7)**, inner nuclear layer **(8)**, inner plexiform layer **(9)**, ganglion layer **(10)**, nerve fiber layer **(11)**, and inner limiting membrane **(12)**. **(**Views **A** and **B;** X 100, **H**&**E**) and **(**Views **C** and **D;** X 400, **H**&**E**)

By LM examinations, the retina in two examined birds resembled that in other birds, consisted of two major layers: the external pigmented epithelium layer and the internal neural layer. The retina is divided into nine layers, including the photoreceptor cell layer, which is composed of cones and rods; the external limiting membrane; the external nuclear layer; the external plexiform layer; the internal nuclear layer; the internal plexiform layer; the ganglion cell layer; the nerve fiber layer; and the internal limiting membrane **(**Fig. 5**)**. The external layer, a single layer of pigmented epithelium, appeared brown due to melanin granules and extended from the outer layer to the photoreceptor layer, supporting and absorbing scattered light that passed through these cells.

The diurnal common kestrel had a thick, dense, externally pigmented epithelium layer in the peripheral region of the photoreceptor layer, while the nocturnal little owl had a scattered pigmented epithelium layer and appeared more a larger and less thick layer of epithelial cells **(**Fig. 5B and D**)** than the diurnal common kestrel **(**Fig. 5A and C**)**. The photoreceptor layer revealed differing types of cones and rods that linked outside with the pigmented epithelium. In the diurnal common kestrel, the visual cell layer is composed of single and double cones, and the external segment of the cones is wide and elongated, while the photoreceptor cell layer in the nocturnal little owl is composed of single elongated rods only. The external segment of the rods was narrow and elongated **(**Fig. 5**).**

**Fig. 6:**
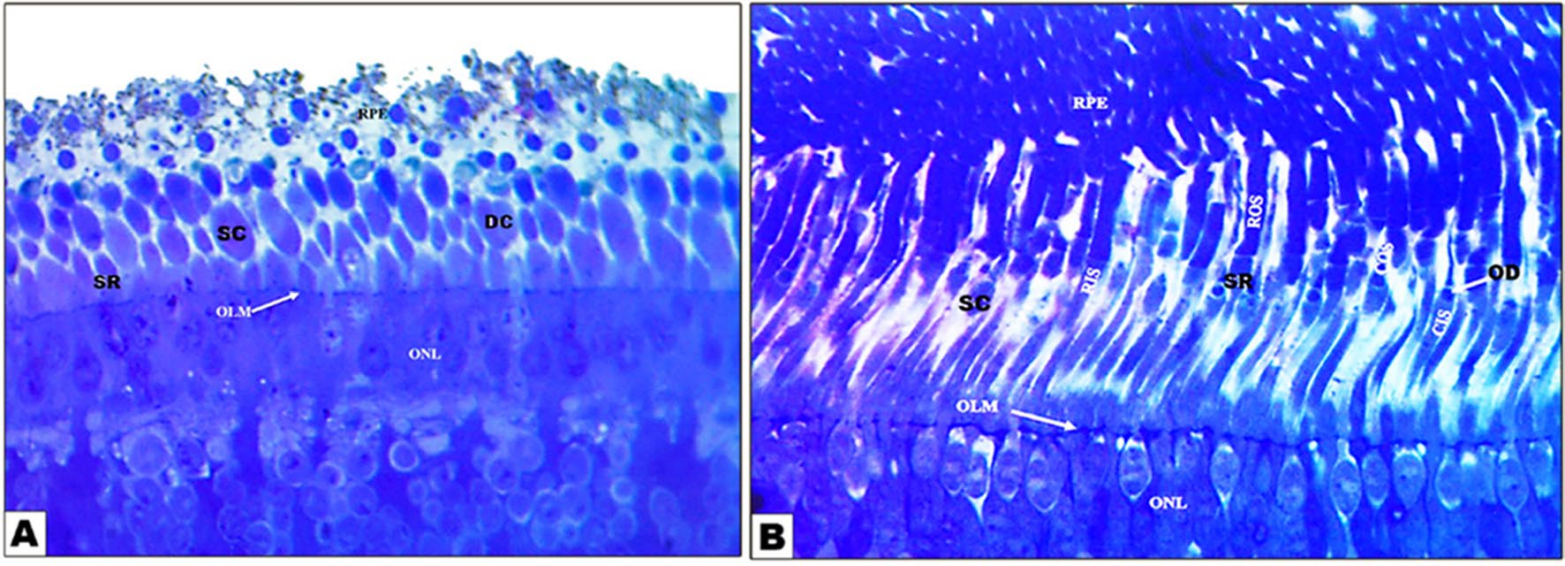
Photomicrograph Semithin section of the rod and cone cells that prepared for Transmission electron examinations of the retina photoreceptor layer of the diurnal common kestrel (View **A**) and nocturnal little owl (View **B**) showing the cytoplasmic process of retinal pigment of epithelial layer (RPE), single cone (SC), double cone (DC), outer nuclear layer (ONL), mall single rod (SR), eouter limiting membrane (OLM), oil droplet (OD), cone outer segment (COS), cone inner segment (CIS), rod outer segment (ROS), and rod inner segment (RIS). (Views A and B X 4000; Views C&D X 8000)

Rod photoreceptors had an outer segment composed of a stack of bimembranous discs enclosed in a limiting membrane **(**Fig. 5**)**. In the light-adapted condition, rod (and cone) outer segments are surrounded by the apical processes of the retinal epithelial (RPE) cells **(**Fig. 5**)**. The RPE in this owl was heavily pigmented more than that in common kestrel, however, and it was doubtful if the amount of pigment present is effective in isolating photoreceptor outer segments from one another **(**Figs. 5 and 6**)**. Cone outer segments may be readily distinguished from rod outer segments by their smaller size and absence of peripheral incisures, which are distributed throughout the latter **(**Fig. 5**)**. Cone outer segments differed from rod outer segments; they were shorter and more conical with a wider base and tapering shape compared with those of rods.

In both examined birds, the outer limiting membranes were a clear light layer that appeared in all areas, and the photoreceptor layer is separated from the external nuclear layer **(**Figs. 5 and 6**).** In both examined birds, the external nuclear layer consisted of the visual cell bodies; the thickness of this layer varied between the two examined bird species. In the diurnal common kestrel, the number of cellular rows was between 4 and 5, while in the nocturnal little owl, it was between 5 and 6 **(**Figs. 5C-D and 6**).** In both examined birds, the external nuclear layer consisted of the visual cell bodies; the thickness of this layer varies between the two studied species. In the diurnal common kestrel, the number of cellular rows was between 4 and 5, while in the nocturnal little owl, it was between 5 and 6 **(**Figs. 5C-D and 6**).**

In both examined birds, the thickness of the external plexiform layer varied, with the diurnal common kestrel having a wide layer with bipolar and horizontal cell densities and the nocturnal little owl having a narrow layer linked to visual cell axons and cell densities of both bipolar and horizontal cells **(**Figs. 5C-D and 6**).** The internal nuclear layer was characterized by its cells, which were compact and diverse and consist of bipolar cells and horizontal cells. This layer varied in thickness between the two examined bird species; in the diurnal common kestrel, this layer was more compact and the number of rows ranged between 14 and 16, while in the nocturnal little owl, this layer and the number of rows ranged between 9 and 11 **(**Figs. 5C-D and 6**).**

In both examined birds, the internal plexiform layer is thicker than the external plexiform layer. This layer consisted of the interlocking of the axes of bipolar cells with the dendrites of the ganglion cells that formed a single layer of the ganglion cell **(**Figs. 5C-D and 6**).** The axons of the ganglion cells were gathered to form the nerve fiber layer, which became thicker as it moved back towards the optic nerve that left the eye and reached the brain. The optic nerve fiber layer is different in thickness in both species **(**Figs. 5C-D and 6**).** The inner limiting membrane separating the retina from the vitreous humor is a base plate of Muller cells **(**Figs. 5C-D and 6**).**

### Transmission electron microscopy (TEM) investigation of retina

**Fig. 7:**
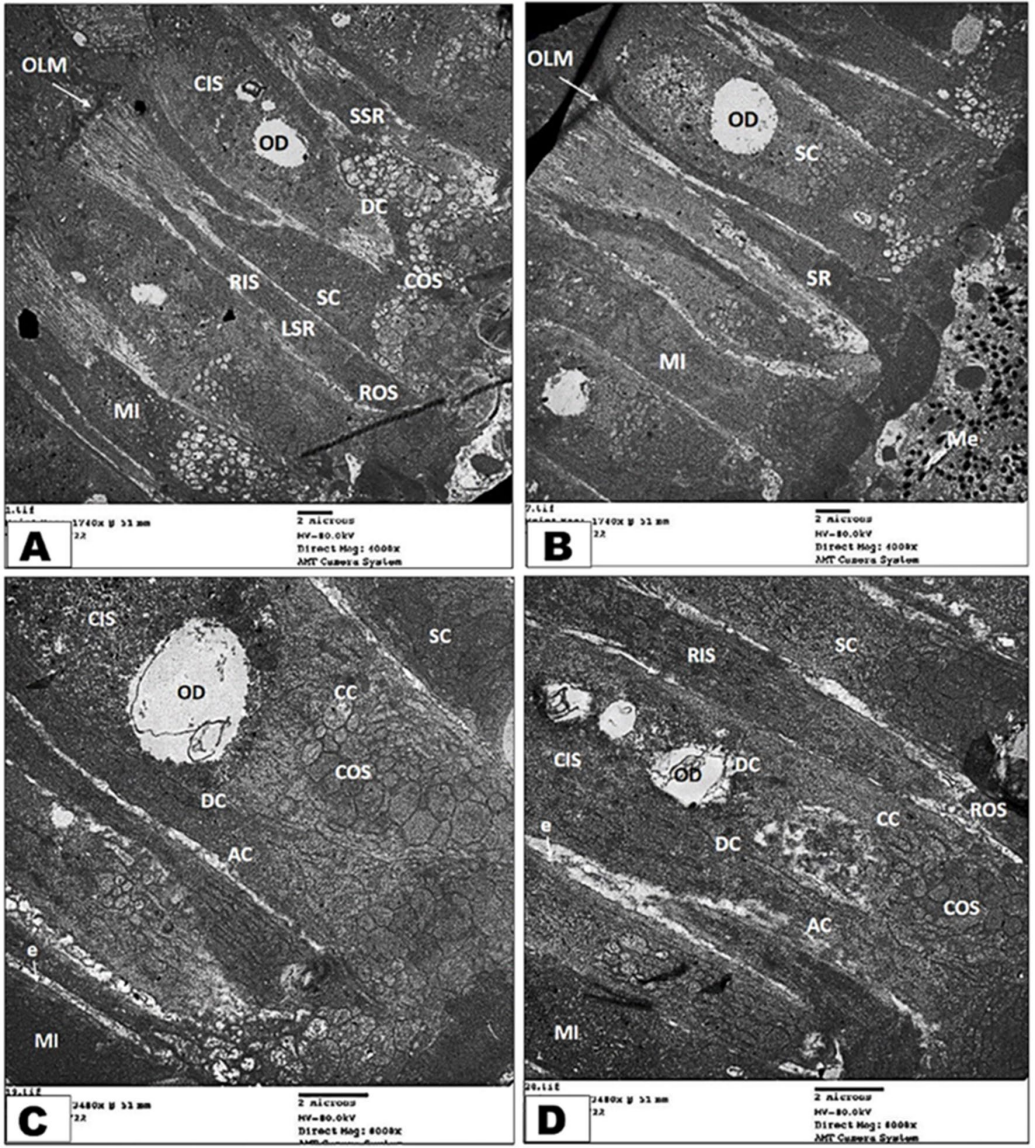
Transmission electron micrograph of the retina photoreceptor layer of the diurnal common kestrel showing the large single rod (LSR), small single rod (SSR), single cone (SC), double cone (DC), melanosomes (Me), ellipsoid region (e), mitochondria (Mi), outer limiting membrane (OLM), oil droplet (OD), cone outer segment (COS), cone inner segment (CIS), chief cone (CC), accessory cone (AC), rod outer segment (ROS), and rod inner segment (RIS). (Views **A** and **B** X 4000; Views **C**&**D** X 8000)

The photoreceptor layer of the retina of the diurnal common kestrel contained the cone-type of photoreceptors with a few types of rod; additionally, their cone cells were categorized as single cone cells (SC), which were large cells (LSC), and double cone cells (DC). The double cone cells were composed of chief cone cells (CC) and accessory cone cells (AC). Both the single and double cone cells consisted of an outer segment (OS) and an inner (IS) segment, which are in association with a few single rod (SR) segments, small and large **(**Fig. 6A-B**)**. The outer segments were closely associated with the pigment epithelial cells **(**Fig. 6C-D**)**, and they were short, wide, and occupied by a membrane as flattened sacs. While the inner segments were very wide, they had densely packed mitochondria in their ellipsoid region and large oil droplets in some of them **(**Fig. 7C-D**).** The single rods had long cylindrical outer segments that reached to the pigments of the epithelial cells **(**Fig. 6D**);** the inner segment of the rod had a large ellipsoid region; but no oil droplets or microdroplets were present. The mitochondria of the rods were less packed and were mostly elongated to oval in shape, in contrast to the cone’s ellipsoid.

**Fig. 8:**
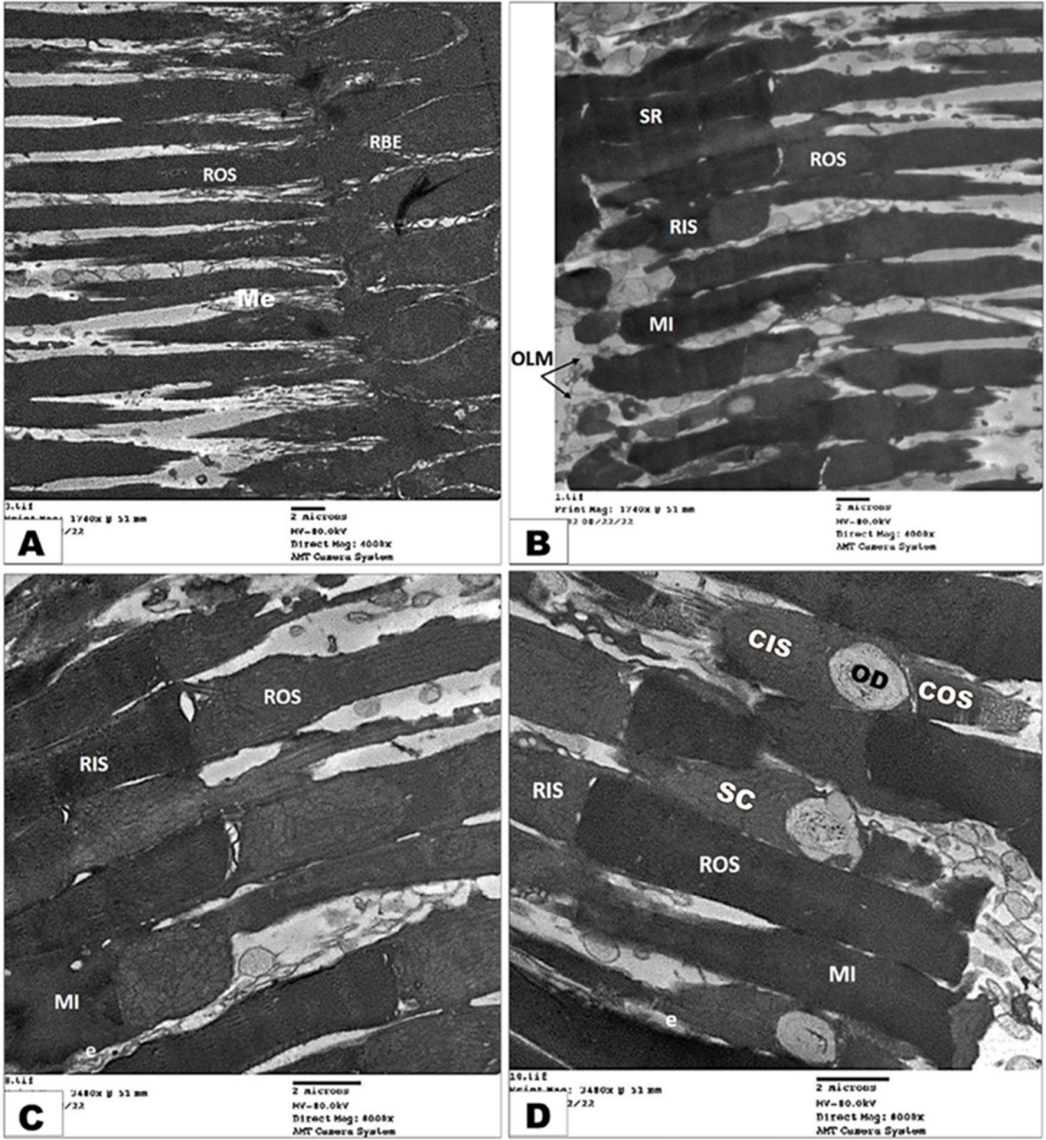
Transmission electron micrograph of the retina photoreceptor layer of the nocturnal little owl showing the melanosomes (Me), ellipsoid region (e), mitochondria (Mi), outer limiting membrane (OLM), cytoplasmic process of retinal pigment of epithelial layer (RPE), single rod (SR), rod outer segment (ROS), rod inner segment (RIS), single cone (SC), cone outer segment (COS), cone inner segment (CIS), and oil droplet (OD). (Views **A** and **B** X 4000; Views **C**&**D** X 8000)

The photoreceptor layer of the nocturnal little owl contained both types of photoreceptors—many rods and a few cones. The rod had one type, single rods, which were very long, cylindrical, and had outer segments that reached to the pigments of the epithelial cells **(**Fig. 8A**)**. The inner segment of the rods had a large ellipsoid region, but no oil droplets or microdroplets were present. While the cone had one type, the single cone differed from the rod in the inner segment, which had lipid droplets, and the outer segment was shorter and more cylindrical **(**Fig. 8B-D**)**.

### Morphometric measurements and statistical comparison analysis between the retinal layer thickness between the diurnal common kestrel and the nocturnal little owl

There was a great variation between the two examined bird species in the thickness of the retinal layers. The pigmented epithelium layer in the diurnal or photopic common kestrel was less thick (76.82 ± 3.21 μm) than that of the nocturnal little owl, which recorded 114.28 ± 2.51 μm. The thickness of the photoreceptor layer in the diurnal common kestrel was 25.56 ± 1.17 μm, which was clearly reduced, compared to the photoreceptor layer of the nocturnal little owl, which was considerably thicker, with an average of 89.84 ± 2.81 μm **(**Table 3**)**.

**Table 3:** Morphometric measurements and statistical comparison analysis between some retinal layer thickness between the diurnal common kestrel and the nocturnal little owl

Neural retina layers (Mean ± SD) µm			
Parameter	Diurnal common kestrel	Nocturnal little owl	*P*-value
PE	76.82 ± 3.21b	114.28 ± 2.51a	*p* < 0.001
PHL	25.56 ± 1.17b	89.84 ± 2.81a	*p* < 0.001
ONL	69.36 ± 3.59a	58.62 ± 1.15b	*p* < 0.01
OPL	30.82 ± 1.61a	17.02 ± 0.73a	*p* < 0.001
INL	214.96 ± 12.46a	144.61 ± 1.29b	*p* < 0.001
IPL	147.80 ± 0.7a	125.80 ± 2.5a	*p* < 0.001
GCL	39.81 ± 3.46a	25.53 ± 1.20a	*p* < 0.001
NFL	197.83 ± 9.41a	60.07 ± 1.59a	*p* < 0.001

Abbreviations: Standard Deviation (SD), Pigment Epithelium (PE), Photoreceptor Layer (PHL), Outer Nuclear Layer (ONL), Outer Plexiform Layer (OPL), Internal Nuclear Layer (INL), Internal Plexiform Layer (IPL), and Ganglion Cell Layer (GCL), Nerve Fiber Layer (NFL). ^a^ and ^b^ indicated the statistical significance with *P* < 0.001

The thickness of the inner nuclear layer and the outer nuclear layer in the diurnal common kestrel (69.36 ± 3.59 μm and 214.96 ± 12.46 μm), respectively, were much larger than those in the nocturnal little owl (58.62 ± 1.15 μm and 144.61 ± 1.29 μm). Also, the thickness of the inner plexiform layer and the outer plexiform layer in the diurnal common kestrel were large compared with those in the nocturnal little owl **(**Table 3**)**. The ganglion cell layer and the nerve fiber layer in the diurnal common kestrel were thicker (39.81 ± 3.45 μm and 197.83 ± 9.41 μm) than those of the nocturnal little owl, which demonstrated the smallest average (25.53 ± 10.20 μm and 60.07 ± 1.59 μm), as illustrated in **(**Table 3**)**.

## Discussion

Our study aimed to compare the visual adaptations of the lens and retinal photoreceptors of two Egyptian endemic carnivorous bird species with different active clock hours using gross, histological, and scanning and transmission electron microscopy techniques. Our findings suggested that the specific types of photoreceptors present in each examined species were directly related to their respective active clock hours. Additionally, the cone-type photoreceptors in diurnal kestrels likely enable them to perceive fine details and colors during daylight hours, while the rod-type photoreceptors in nocturnal little owls enhance their ability to detect low levels of light in dimly lit environments.

In comparison to other vertebrates [6], birds have the most highly developed sensory vision system [44–46]. Compared to the size of their heads, the bird eyes are large, and if compared to animal eyes, they are substantially bigger [38, 47, 48]. The eyes of owls and eagles are comparable to or bigger than those of animals, while those of the common ostrich are around twice as large [49]. The eyes of many bird species, including songbirds, are almost the same size as their brains [50]. The current findings reveal that the eyeball of the diurnal common kestrel is semi-spherical, with less convexity on the anterior surface than on the posterior surface; meanwhile, in the nocturnal little owl, the eyeball is relatively larger than the eyeball of the diurnal common kestrel, with elongated eye tubes or cylinders in the shape and an end with a flattened disc. Our findings confirm that these adaptations in the little owl’s eyeball allow for enhanced light-gathering ability, which is crucial for its nocturnal hunting behavior; additionally, the flattened disc at the end of the eye tubes helps to improve depth perception, aiding in precise prey targeting during low-light conditions.

Our findings agrees with previously published data that the main structures of the bird eye are similar to those of other vertebrates [6, 7, 18, 51]. Consistent patterns of variation in eye shape in relation to activity patterns and habitats have been reported across vertebrates [6, 10, 28, 47, 52], including birds [18, 44, 53–55]. The ratio of corneal diameter (CD) to eye axial length (AL), which is used here to characterize eye shape, differs with different activity patterns in terrestrial vertebrates, such as birds and reptiles [55–57]. Our findings on the nocturnal little owl agrees with that previously reported that the majority of nocturnal species that are active in low-light conditions have comparatively bigger corneas and high CD: AL ratios, so greater visual sensitivity is achieved by increasing the amount of photons that reach the retina through a larger pupil, which is made possible by a larger cornea [38, 53]. However, our findings on the diurnal kestrel agrees with that previously reported that the diurnal bird species, do not depend on the availability of light, and as a result, their eyes have low CD: AL ratios and, consequently, significantly greater AL and focal lengths. This eye shape is associated with improved spatial resolution.

Recently, a diverse sample of over 450 bird species showed that there are significant differences in eye shape among birds with nocturnal and diurnal activity patterns [38]. Our findings describes the relationship between corneal diameter (CD) and axial length (AL) of the eye, in which the diurnal common kestrel has a noticeably smaller cornea diameter (CD) relative to eye axial length (AL), which has the lowest CD: AL ratios of -0.175. In contrast, the nocturnal little owl occupies one end of the spectrum with the largest cornea diameter (CD) relative to eye axial length (AL) and an average log 10 (CD: AL) ratio of -0.109. Functionally, our study confirms that these differences reflect differences in the light-gathering capabilities of eyes that operate under luminance levels that vary by about 12 log units. These findings come in contact with those reported in some birds [58–60]. Additionally, the average CD: AL ratios for the diurnal raptors are (-0.175 ± 0.013) and the nocturnal raptors (-0.109 ± 0.011), as described by **Hall and Ross** [38].

Our findings reveal the first statistical analysis in both birds: the axial length of the eye and the corneal diameter are highly correlated. The Pearson correlation coefficient of 0.91 in the diurnal common kestrel indicates a strong positive correlation between axial length (AL) and corneal diameter (CD), indicating an increase in both. In the nocturnal little owl, the Pearson correlation coefficient is 0.89, also indicating a strong positive correlation between AL and CD. Like in the kestrel, a longer axial length corresponds to a larger corneal diameter. Meanwhile, the nocturnal little owl has a strong positive correlation between AL and CD, with longer axial length indicating larger corneal diameter, similar to the kestrel.

Our findings reveal that the eye lens of the diurnal common kestrel is less convex on the anterior surface than the posterior one, similar to that described in the diurnal ostrich [61], whereas in the nocturnal little owl, the convexity of the lens on its anterior surface is greater than the posterior one. The same state is also shown in the eye lenses of other animals [62], while in some animals, including rodents and marine mammals, the lens is round [63]. Our findings illustrate that the average diameter of the diurnal kestrel lens is 0.5 ± 0.14 cm, and its average thickness (anterior-posterior) is 0.42 ± 0.075 cm; meanwhile, the average diameter of the nocturnal little owl lens is 1.1 ± 0.26 cm, and its average thickness (anterior-posterior) is 0.52 ± 0.12 cm. However, in the ostrich, the lens diameter is 1.43 ± 0.00 cm and its mean anterior-posterior thickness is 0.85 ± 0.00 cm [61], while the chicken had a lens diameter of about 0.65 cm and an anterior-posterior thickness of about 0.40 cm [62]. Our research reveals that the lens diameter and thickness in the little owl were greater than those in chickens and are the same as those in ostriches [61]. Functionally, our findings suggest that lens size may be correlated with the visual requirements and adaptations to the environment in which they live, in which the larger lens diameter and thickness in ostriches and little owls may be indicative of their need for enhanced visual acuity or specialized visual capabilities. Functionally, our study confirms that these differences in lens convexity suggest that the common kestrel relies more on its visual acuity for hunting during the day, while the little owl may prioritize light-gathering ability for nocturnal activities. Additionally, these variations in lens shape could be an adaptation to optimize vision in different lighting conditions and hunting strategies.

Like most animals, the bird’s eye lens is composed of three components: the capsule, the epithelium cells, and the lens fibers. The capsule completely encloses the eye lens and contains basal membrane components that have been replaced by a sheet or many layers of collagen fibrils [64]. The current histological observations show that the lens of the examined diurnal common kestrel is covered by a thin capsule, and the outer part of the lens capsule is less dense and consists of fine fibers. Meanwhile in the nocturnal little owl, the lens surface is surrounded by the outermost layer of a dense thick capsule, which seems to have a hexagonal shape of fibers. The dense thick capsule in the nocturnal little owl is also observed in the ostrich’s eye [61]. Also, our histological study shows squamous epithelial cells with round nuclei surrounding light cytoplasm, which is reported in the ostrich [61]. Moreover, the lens fibers under the capsule are observed to be parallel to each other, and in the examined nocturnal little owl, they seem to be thicker in the lateral areas than the middle ones, as in the eye lens of the ostrich [61]. In other animals, on the lateral part of the lens, the epithelial cells become longer and form the fibers in the body of the lens after differentiation [65]. The cuboidal cells that make up the epithelium of the lens are arranged in a layer, with their tops facing the lens fibers and their bottoms facing the lens capsule [64]. In non-mammal animals, the lens has cylindrical radial fibers, which help the lens change its shape for accommodation [63]. Functionally, the epithelium cells are responsible for the production and maintenance of the lens fibers, which are long, transparent proteins that give the lens its shape and clarity. These fibers are arranged in a highly organized pattern that allows the lens to focus light onto the retina, enabling birds to have sharp vision.

There are differences in the anatomy of the retina, the regions of highest visual acuity, and retinal vascularization in birds [19], despite the fact that the organization of retinal layers in birds is the same as that in other vertebrates [6, 47]. Our histological findings agree with those described in all animals, including birds [6, 10, 18, 28, 47, 66], that the retina consists of two major layers: the external pigmented epithelium layer and the internal neural layer; additionally, the retina consists of nine layers, including the photoreceptor cell layer, which is composed of cones and rods; the external limiting membrane; the external nuclear layer; the external plexiform layer; the internal nuclear layer; the internal plexiform layer; the ganglion cell layer; the nerve fiber layer; and the internal limiting membrane.

The current findings in both examined birds, the external single brown pigmented epithelium layer of the retina is due to the presence of the melanin granules and extends from the outer layer to the photoreceptor layer, supporting and absorbing scattered light that passes through these cells. Our findings reveal that the diurnal common kestrel has a thick, dense, externally pigmented epithelium layer in the peripheral region of the photoreceptor layer, while the nocturnal little owl has a scattered pigmented epithelium layer and appears as a larger and less thick layer of epithelial cells than the diurnal common kestrel. The presence of the melanin granules is also reported in most birds [18], and they added that because the Garganey is a nocturnal and diurnal bird, its retinal pigment epithelium (RPE) has a more powerful pigmentation because melanin provides protection against ultraviolet radiation. The retinal pigment epithelium (RPE) plays crucial functions such as modifying and storing vitamin A, facilitating material passage through photoreceptor cells, stabilizing and orienting photoreceptor cells, preventing retinal detachment, and dehydrating and regulating subretinal space, as described in most birds [67–69]. Functionally, the highly melanized RPE observed in vertebrate retinas contributes significantly to the absorption of extra light before it reaches the photoreceptors [23].

The powerful avian visual systems are comparatively well studied, and the principles of bird vision depend on their cone and rod photoreceptor cells. Most birds have rod photoreceptors for dim-light vision and cone photoreceptor types for color vision [31]. Our findings in the diurnal kestrel agree with those described in the retina of both diurnal and nocturnal activity birds [19, 66, 70–73], that there are cone and rod cells, with the cones more than rods, but in the nocturnal Eurasian Eagle-owl, the photoreceptor layer of the retina contains simple cones and rods [66]. In diurnal vertebrates, the ratio of cones to rods favors the retina’s ability to respond quickly to incoming light as well as to a wide range of light intensities [74, 75], while the rods in the same retina respond to light more slowly but more sensitively than the cones, and they can function in conditions of very low light [74, 76]. Our findings illustrate that the photoreceptor (visual cell) layer in diurnal common kestrel is composed of single and double cones, and the external segment of the cones is wide and elongated; meanwhile, the photoreceptor cell layer in nocturnal little owl is composed of single elongated rods only, and the external segment of the rods is narrow and elongated. Rods are more sensitive to light but do not provide color information [51], whereas less sensitive cones provide color vision. In nocturnal owls, practically all of the receptors are rods, whereas 80% of the receptors in diurnal birds may be cones (90% in certain swifts) [66]. **Montoyo**, et al. [31] reported that the booted eagle has a single class of rods, a single kind of double cone, and numerous varieties of single cones that are distinguished by the presence of oil droplets at the distal end of their inner segments.

Our findings report that the internal nuclear layer (INL) is characterized by its cells, which are compact and diverse and consist of bipolar cells and horizontal cells. This layer varies in thickness between the two examined bird species; in the diurnal common kestrel, this layer is more compact and the number of rows ranges between 14 and 16, while in the nocturnal little owl, this layer and the number of rows range between 9 and 11. Our findings agree with the previously published findings that, in comparison to the other retinal cell layers, the inner nuclear layer of carnivorous birds was comparatively thicker [75, 77]. Functionally, the presence of a high density of retinal neural cells, which are important for photo transduction, is the reason why a bird’s INL is relatively thicker than that of other vertebrates. Our findings report that the thicknesses of the INL, ONL, IPL, and OPL in the examined diurnal kestrel are thicker than those in the nocturnal little owl. They have also observed that the thickness of the INL and IPL in the retina of the booted eagle presents a high degree of complexity in processing and neural interactions [31].

Our transmission electron findings reveal that the photoreceptor layer of the retina of the diurnal common kestrel contains the cone-type of photoreceptors with a few types of rod; additionally, their cone cells were categorized as single large cone cells and double cone cells, which are composed of chief cone cells and accessory cone cells, similar to those reported in other diurnal birds [71, 75]. Meanwhile, the nocturnal little owl has only rod-type photoreceptors, similar to those reported in other nocturnal birds [23]. Moreover, our transmission electron findings reveal that both the single and double cone cells consist of an outer segment and an inner segment, which are in association with a few single rod segments, small and large. The outer segment of cone cells of the diurnal common kestrel is closely associated with the pigment epithelial cells, while the inner segments have densely packed mitochondria in their ellipsoid region and large oil droplets in some of them. Additionally, because the melanosomes do not directly contact a photoreceptor, they play a special role in light absorption by preventing light scattering and the subsequent decline in visual acuity [78]. Moreover, McBee, et al. [79] found that the melanin-containing organelles (melanosomes) serve a crucial role in protecting RPE cells from negative effects that may arise during processes of photopigment recycling and phagocytosis, as well as in reducing the amount of light that is reflected back into the eye.

Our transmission electron findings reveal that the mitochondria of the cones and rods are less packed and are mostly elongated to oval in shape, in contrast to the cones ellipsoid. Our transmission electron findings reveal that the single rods of the diurnal common kestrel have long cylindrical outer segments that reach to the pigments of the epithelial cells; meanwhile, in the only rod-type photoreceptor cells of the nocturnal little owl, the inner segment has a large ellipsoid region, but no oil droplets or microdroplets are present. The outer segments of the photoreceptors serve as the photoreceptors’ light capture field [24], while **El-Beltagy** [75], **Cohen** [80] added that the cone’s outer segment is shorter and distally tapered compared to the rod’s long, constant-diameter outer segment.

Our transmission electron findings reveal that the single rods of both examined bird species have an inner segment that has a large ellipsoid region, but the little owl has a cone with an oil droplet. These results agree with **Alix**, et al. [66], **Bowmaker and Martin** [81], **Gondo and Ando** [82], **Hart and Hunt** [83], who mentioned that nocturnal or dim-light active bird species have very pale or colorless cone oil droplets. In the inner segments of the cones of vertebrates, there are organelles known as ellipsoids, which are collections of mitochondria [84]. Even animals with oil droplets, like birds, have these ellipsoids [23, 75, 80, 85, 86]. As in other vertebrates, an aggregation of mitochondria (ellipsoid) may be seen near the apex of the inner segments of the various photoreceptor cells. The distribution and direction of these mitochondria vary depending on the photoreceptor. In nocturnal species, it has been hypothesized that this area would function as a convex lens, concentrating light on the outer segment and improving visual acuity; however, in nocturnal animals, this is not essential [75, 86].

## Conclusion

Our study aimed to compare the anatomical visual features of the eyeball, lens, and retina of the diurnal common kestrel and the nocturnal little owl between the two carnivorous birds with different active clock hours. The semi-spherical eyeball of the kestrel had a less convex anterior surface than the posterior surface; meanwhile, it was relatively larger in the owl. The retinal thickness in the kestrel had a thinner pigmented epithelium and photoreceptor layers compared to those in the nocturnal owl. The photoreceptor layer in diurnal kestrel had single and double cones (single large cones and double cones); meanwhile, the nocturnal owl had only single elongated rods. The statistical analysis in both birds: the axial length of the eye and the corneal diameter are highly correlated. The Pearson correlation coefficient of both birds indicated a strong positive correlation between axial length (AL) and corneal diameter (CD).

## Data Availability

The datasets used and/or analyzed during the current study are available from the corresponding author on reasonable request.
